# Effects of sacubitril-valsartan on heart failure patients with mid-range ejection fractions: A systematic review and meta-analysis

**DOI:** 10.3389/fphar.2022.982372

**Published:** 2022-10-24

**Authors:** Jianbin Qin, Weijian Wang, Ping Wei, Ping Huang, Ruizhen Lin, Jinming Yue

**Affiliations:** Department of Cardiology, Wuzhou Red Cross Hospital, Wuzhou, China

**Keywords:** sacubitril-valsartan, median ejection fraction, heart failure, systematic review, meta-analysis

## Abstract

**Aim:** The effect of sacubitril-valsartan (ARNI) in heart failure (HF) patients with mid-range ejection fractions (HFmrEF) remains unclear. This study aimed to investigate the effects of ARNI in HFmrEF patients.

**Methods:** From inception to 15 February 2022, articles were searched *via* PubMed, Embase, Cochrane Library, Web of Science, China National Knowledge Infrastructure, Whip, and Wanfang databases. Left ventricular functions, indicators related to HF, quality of life score, 6-Minute Walk Test, total effective rate, mortality, readmission rate, and adverse events were the outcomes. Relative risk (RR), weighted mean difference (WMD), and 95% confidence interval (CI) were used to evaluate the outcomes. The heterogeneity test was conducted for each indicator and measured by I^2^ statistics. Subgroup analysis was performed regarding the type of study and duration of treatment.

**Results:** Sixteen studies involving 1,937 patients were included in this study. Our results showed ARNI was likely to improve left ventricular function by increasing the left ventricular ejection fraction (LVEF) (WMD: 2.36, 95%CI: 1.09–3.62), stroke volume (WMD: 16.800, 95%CI: 11.385–22.215), and left ventricular short-axis shortening rate (WMD: 2.05, 95%CI: 0.25–3.86), decreasing left ventricular end-diastolic dimension (WMD: −2.48, 95%CI: −3.83 to −1.13), left atrial diameter (WMD: −2.23, 95%CI: −2.83 to −1.63), C-reactive protein level (WMD: −1.40, 95%CI: −2.62 to −0.18), and N-terminal-pro B-type natriuretic peptide level (WMD: −494.92, 95%CI: −641.34 to −348.50). ARNI has a higher total effective rate (RR: 1.15, 95%CI: 1.08–1.21), Kansas City cardiomyopathy questionnaire (WMD: 4.13, 95%CI: 3.46–4.81), and 6-Minute Walk Test (WMD: 51.35, 95%CI: 26.99–75.71) compared with angiotensin-converting enzyme inhibitors (ACEI) and angiotensin receptor blockers (ARB). In addition, ARNI decreased the readmission rate (RR: 0.54, 95%CI: 0.43–0.68) (all *p* < 0.05). Nevertheless, there were no significant differences in the adverse outcomes.

**Conclusion:** This meta-analysis suggests ARNI may be an effective strategy with which to improve the left ventricular function, and quality of life, and reduce the readmission rate in HFmrEF patients. However, long-term clinical studies with large samples are still needed to further explore the efficacy and safety of ARNI compared with ACEI or ARB in the HFmrEF population.

## Introduction

Heart failure (HF) is a clinical syndrome that results from any structural or functional impairment of ventricular filling or ejection of blood (Bozkurt et al., 2021). HF with mid-range ejection fraction (HFmrEF) with left ventricular ejection fraction (LVEF) ranging from 41 to 49% is a category of HF (McDonagh et al., 2022). HFmrEF is a hemodynamic state in which the heart cannot meet the circulatory demands of the body, or at the expense of increased left ventricular filling pressure (Ye et al., 2022). The incidence of HFmrEF accounts for 10–20% of the population with HF (Ponikowski et al., 2016a; Srivastava et al., 2020). HFmrEF is associated with notable morbidity and mortality (Rickenbacher et al., 2017). Consequently, it is essential to find therapies for HFmrEF patients.

Substantial evidence has indicated that angiotensin-converting enzyme inhibitors (ACEI) and angiotensin receptor blockers (ARB) could improve the partially attenuate left ventricular (LV) dilation and remodeling in HF, however, the morbidity and mortality of HF patients remain unacceptably high (Asgar et al., 2015; Zhang et al., 2020). In recent years, angiotensin receptor neprilysin inhibitor (ARNI) also named sacubitril-valsartan demonstrated to reduce mortality and morbidity of HF and is now a new recommended treatment option for symptomatic reduced ejection fraction (HFrEF) according to the recommendations from the American College of Cardiology (ACC), and the European Society of Cardiology (ESC) (Maddox et al., 2021; McDonagh et al., 2022). ARNI mainly focuses on inhibiting the activity of neprilysin to decrease the degradation of natriuretic peptides, stimulate vasodilation and diuresis, and reduce myocardial fibrosis and hypertrophy, which has shown clinical benefits in HF with HFrEF (Pascual-Figal et al., 2021). Nevertheless, there are currently limited studies comparing the effects of ARNI and traditional ACEI/ARB drugs on patients with HFmrEF. There are differences in LVEF, epidemiological characteristics, and pathogenesis of patients with different types of HF, so it is necessary to seek optimal treatment for HFmrEF patients (Xin et al., 2019). Furthermore, the efficacy and safety of ARNI in patients with HF are still controversial (Zhang et al., 2020). Hence, a meta-analysis to assess and compare the ARNI and traditional ACEI/ARB drugs on HFmrEF patients is needed.

Herein, we performed a meta-analysis to evaluate the potential clinical benefits and safety of ARNI in HFmrEF patients in which ARNI was compared with ACEI/ARB drugs.

## Methods

This meta-analysis was performed based on the Preferred Reporting Items for Systematic Reviews and Meta-Analyses guidelines without registry.

### Data sources and search strategy

Databases including PubMed, Embase, Cochrane Library, Web of Science, China National Knowledge Infrastructure, Whip, and Wanfang were searched without any restrictions from inception to 15 February 2022. The search strategy from the PubMed consisted of the following keywords: “Sacubitril Valsartan’’ OR “sacubitril and valsartan sodium hydrate drug combination’’ OR “sacubitril valsartan sodium hydrate’’ OR “sacubitril-valsartan sodium hydrate drug combination’’ OR “trisodium (3-(1-biphenyl-4-ylmethyl-3-ethoxycarbonyl-1-butylcarbamoyl)propionate-3′-methyl-2′-(pentanoyl(2′-(tetrazol-5-ylate)biphenyl-4′-ylmethyl)amino)butyrate) hemipentahydrate’’ OR “sacubitril and valsartan drug combination’’ OR “sacubitril valsartan drug combination’’ OR “sacubitril-valsartan OR 3-(1-biphenyl-4-ylmethyl-3-ethoxycarbonyl-1-butylcarbamoyl) propionate-3′-methyl-2′-(pentanoyl(2′-(tetrazol-5-ylate)biphenyl-4′-ylmethyl)amino) butyrate’’ OR “sacubitril and valsartan sodium anhydrous drug combination’’ OR “sacubitril valsartan sodium anhydrous’’ OR “sacubitril-valsartan sodium anhydrous drug combination’’ OR “LCZ 696’’ OR “LCZ696’’ OR “LCZ-696’’ OR “Entresto’’ AND “Heart failure’’ OR Cardiac Failure’’ OR “Heart Decompensation’’ OR “Decompensation, Heart’’ OR “Heart Failure, Right-Sided’’ OR “Heart Failure, Right Sided’’ OR “Right-Sided Heart Failure’’ OR “Right Sided Heart Failure’’ OR “Myocardial Failure’’ OR “Congestive Heart Failure’’ OR “Heart Failure’’, Congestive OR “Heart Failure, Left-Sided’’ OR “Heart Failure, Left Sided’’ OR “Left-Sided Heart Failure’’ OR “Left Sided Heart Failure’’ OR “Heart failure with mid-range ejection fraction’’ OR “HFmrEF’’ OR “HFmEF’’.

### Selection criteria

Inclusion criteria based on the PICOS principles were: 1) Populations: Patients diagnosed with HFmrEF; 2) intervention: ARNI as the observation group; 3) comparators: ACEI or ARB as the control group; 4) outcomes: left ventricular function, indicators related to HF, quality of life score, 6-Minute Walk Test (6-MWT), total effective rate, mortality, readmission rate, and adverse events; 5) study design: Randomized controlled trials (RCTs) or cohort studies 6) studies published in English and Chinese.

The exclusion criteria were as follows: 1) animal experiments; 2) case reports, meta-analyses, reviews, and letters.

### Data extraction and quality assessment

Two investigators (Jianbin Qin and Weijian Wang) initially screened studies based on abstracts and reviewed the full text according to eligibility criteria. The final qualification for inclusion depended on the agreement between the two reviewers. Any differences should be resolved through consultation with the third reviewer (Ping Wei). The following data were extracted from each included study: basic characteristics of studies (authors, publication year, country, study design), characteristics of patients (sample size, gender, age), ARNI and ACEI/ARB treatments (dosage and duration of treatment), outcomes, and quality score of include studies.

For cohort studies, the quality of the literature was evaluated using the modified Newcastle-Ottawa criteria scale (NOS) (Stang, 2010). The total score of the scale was 10, with <5 as low quality and ≥5 as high quality. RCT was evaluated by the modified Jadad rating scale, in which 1 to 3 and 4 to 7 were considered as low and high quality, respectively (total scores: 7) (Jadad et al., 1996).

### Variables and outcomes assessment

According to ESC guidelines, the diagnostic criteria for HFmrEF in this study was a LVEF of 40–49% (Ponikowski et al., 2016b).

The primary outcomes were left ventricular functions including left ventricular ejection fraction (LVEF), left ventricular end diastolic dimension (LVEDD), left ventricular end-systolic diameter (LVESD), diastolic ventricular septal thickness (DVST), left atrial diameter (LAD), stroke volume (SV), left ventricular short axis shortening rate (FS), C-reactive protein (CRP) level. The second outcomes were 1) indicators related to HF including N-terminal-pro brain natriuretic peptide (NT-proBNP), soluble suppression of tumorigenesis-2 (sST2), growth differentiation factor-15 (GDF-15); 2) quality of life score including Minnesota heart failure scale score (MHFQL), and The Kansas city cardiomyopathy Questionnaire (KCCQ); 3) 6-MWT; 4) total effective rate; 5) mortality including 1-year mortality, and cardiac death; 6) readmission rate; 7) adverse events including HF worsen, malignant arrhythmia, renal function deterioration, hyperkalemia, hypotension, angioedema, serum creatinine (SCr) level. LVEF was calculated as end-diastolic minus end-systolic volume divided by end-diastolic volume (Panza et al., 2019). The normal range of LVEDD was 35–56 mm, LVESD was 20–40 mm, and LAD was 27–40 mm (Yang et al., 2018). Quality of life was assessed by the MHFQL and KCCQ. MHFQ is a 21-item disease specific instrument with scores varying from 0 to 5 and a summary score varying from 0 to 105, the highest score representing the worst health-related quality of life (Napier et al., 2018). The KCCQ was a 23-item, self-administered questionnaire that quantifies physical function, symptoms, social function, self-efficacy, and quality of life for patients with HF, The higher the score, the better the quality of life (Green et al., 2000). The total effective rate was assessed in 3 outcomes: 1) significantly effective: after treatment, the symptoms of HF were significantly improved, the LVEF was significantly increased, and the cardiac function grade was decreased to >2; 2) effective: after treatment, the symptoms of HF were relieved, and LVEF was reduced, and the cardiac function grade has decreased by >1; 3) no curative effect: after treatment, the symptoms of HF and LVEF did not improve or even worsened (Xiangjie Liu, 2021). Readmission rate referred to the ratio of hospital readmission due to HF to follow-up. Hypotension was defined as blood pressure < 90/60 mm Hg (McMurray et al., 2014). Deterioration of renal function was defined as a relative increase of serum creatinine ≥25% or an increase of serum creatinine ≥0.3 mg/dl (1 mg/dl = 88.4 μmol/L) from baseline (Bhatt et al., 2018). Hyperkalemia referred to blood clear potassium > 5 mmol/L during the follow-up period (Akinlade et al., 2014). The angioedema was angioneurotic edema, also known as giant urticaria, which involves deep layers of skin, including subcutaneous tissues, as well as airway mucosa, and is manifested as localized non-pitting edema occurring in local tissues (Zuberbier et al., 2018).

### Statistical analysis

Relative risk (RR) was used as an effect indicator for categorical data. Continuous data were analyzed by calculating weighted mean difference (WMD), and the effect size was expressed by 95% confidence intervals (CIs). Heterogeneity of effects across trials was evaluated by I^2^ tests for heterogeneity. When the heterogeneity statistic I^2^ ≥ 50%, random-effects model analysis was performed, otherwise, fixed-effects model analysis was applied. *p* < 0.05 was considered statistically significant. When the difference was statistically significant and I^2^ ≥ 50%, subgroup analysis was performed regarding the type of study and duration of treatment. Sensitivity analysis was performed for all outcomes. Begg’s test was examined to evaluate the potential for publication bias. When publication bias occurred, the “cut-and-fill method” was adopted to adjust publication bias. Software Stata 15.1 (Stata Corporation, College Station, TX, United States) was used for statistical analysis.

## Results

### Literature search and characteristics of studies

**FIGURE 1:**
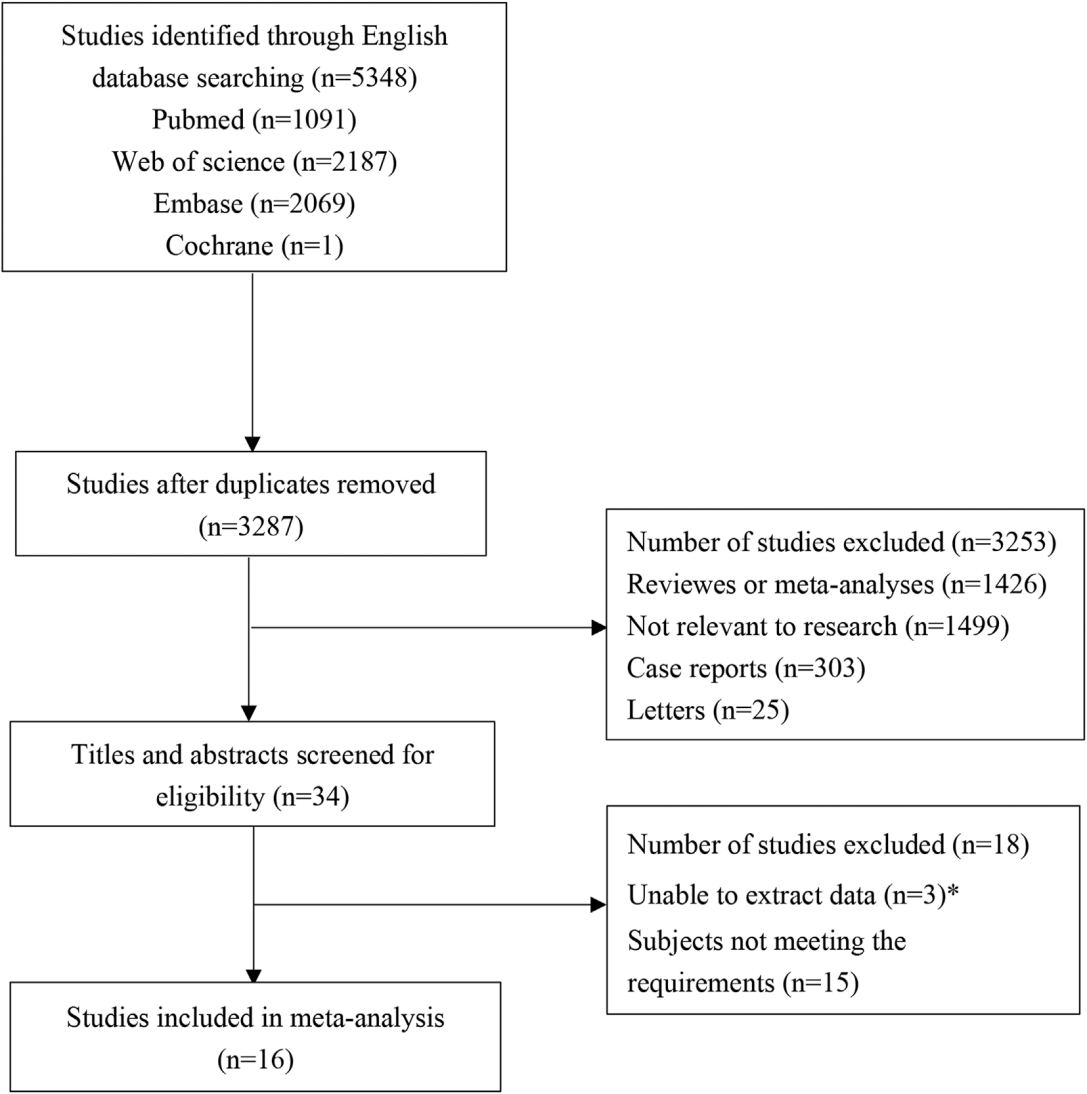
Flow chart of study selection and patient’s collection.

A total of 5,408 studies were identified in the initial literature search. By removing duplicates, 3,287 articles were retrieved. After screening for titles and abstracts, 34 articles were left. Finally, 16 studies (Wang, 2019; Cunfang Chen et al., 2020; He Wen et al., 2020; Pengfei Ma, 2020; Rong Liu et al., 2020; Dongruil Xu and Liu, 2021; Man Gao et al., 2021, Meixian Chen et al., 2021; Mi, 2021; Wu Yi et al., 2021; Xiangjie Liu, 2021; Xinxin Guo, 2021; Yongyue Zhou, 2021; Xiang Li, 2021; Ye et al., 2022; Lr Liana Tumasyan et al., 2019) were included in this study, involving 6 cohort studies and 10 RCTs. A total of 1,937 patients participated in the study, including 913 in the experimental group and 1,024 in the control group. There were 3 low-quality articles and 13 high-quality articles included. The flow chart of study selection is shown in Figure 1. The basic characteristics of included studies are presented in Table 1. The data of each outcome before and after treatment between the observation group and control group are presented in Table 2.

**TABLE 1:** Basic characteristics of included studies.

Author	Year	Country	Study design	Groups	Drugs	Duration of treatment (months)	Sample size [*n* (%)]	Age (years, mean ± SD)	Male/Female [*n* (%)]	Diabetes	Outcomes	Quality
Wang	2019	China	RCT	ARNI	Sacubitril valsartan, the initial dose is 50 mg once, bid; in the later stage, it is increased according to the patient’s tolerance, specifically every 2–4 weeks, until it reaches a stable 200 mg once, bid	3	48	55.9 ± 5.4	35/13	NA	NT-proBNP, LVEF, Scr, total effective rate	5
Tumasyan	2019	Armenia	RCT	ACEI/ARB	ACEI/ARB	12	48	57.5 ± 4.3	37/11	NA	1-year mortality, readmission rate	3
				ARNI	Sacubitril/valsartan, 200 mg, bid		27	59.9	NA	NA		
				Ramipril	Ramipril 10 mg/Valsartan 160 mg, bid		55	59.9	NA	NA		
Liu	2020	China	Retrospective cohort	ARNI	Sacubitril valsartan, from the minimum dose of 25.0 mg/time, orally bid, gradually increased to the maximum tolerated dose of 200.0 mg/time, orally bid	1	20	61.75 ± 13.04	16/4	NA	NT-proBNP, LVEF, LVESD, LAD	4
				Benazepril	Benazepril hydrochloride, from the minimum dose of 2.5 mg/time, orally once a day, gradually increased to the maximum tolerated dose of 10 mg/time, orally once a day		20	60.50 ± 13.47	17/3	NA		
Wen	2020	China	RCT	ARNI	Sacubitril valsartan, 100 mg, bid	6	41	55 ± 7	28/13	11	Total effective rate, NT-proBNP, GDF-15, sST2, LVEF, FS, 6-MWT, quality of life, readmission rate, adverse events	4
				Valsartan	Valsartan, 80 mg, bid		41	53 ± 6	25/16	8		
Ma	2020	China	Retrospective cohort	ARNI	Sacubitril valsartan, the initial dose is 50 mg/time, bid, and after 2–4 days, it is increased by one time according to the patient’s tolerance, until the target dose of 200 mg/time, bid	3	50	64.58 ± 8.39	26/24	NA	Total effective rate, NT-proBNP, LVEDD, DVST, LAD, LVEF, quality of life	5
				Analapril	Analapril tablets, 5 mg/time, bid		50	64.37 ± 8.45	28/22	NA		
Chen	2020	China	RCT	ARNI	Sacubitril valsartan, the initial dose is 25 mg, bid, and the dose is doubled every 2–4 weeks, and the dose is gradually titrated to the individual maximum tolerated dose according to the patient’s blood pressure tolerance, or the target dose, 200 mg, bid	6	53	72.3 ± 10.1	24/29	22	NT-proBNP, LVEF, LVEDD, cardiac death, readmission rate	5
				ACEI/ARB	ACEI/ARB		53	69.5 ± 9.6	31/22	18		
Mi	2021	China	RCT	ARNI	Sacubitril valsartan, 25 mg/time, bid; then gradually increased to 200 mg/time, bid	1	30	64.22 ± 11.52	19/11	NA	LVEF, LVESD, LVEDD	4
				Benazepril	Benazepril, the initial dose is 2.5 mg/time, qd; then the dose is gradually increased to 10 mg/time, qd		30	63.15 ± 11.37	17/13	NA		
Xu	2021	China	RCT	ARNI	Sacubitril valsartan, 50 mg, bid, according to the disease and tolerance, the dose is doubled every 2–4 weeks until 100 mg, bid	6	49	66.6 ± 13.6	32/17	11	NT-proBNP, sST2, CRP, SCr, LVEDD, DVST, LAD, LVEF, 6-MWT, readmission rate, cardiac death, adverse events	6
				Eenalapril	Eenalapril, 10 mg, bid		49	69.4 ± 12.8	27/22	15		
Gao	2021	China	Prospective cohort	ARNI	Sacubitril valsartan, start with a low dose of 50 mg and gradually increase to a target dose of 200 mg according to the patient’s condition and blood pressure tolerance, bid	12	43	61 ± 11	22/21	7	NT-ProBNP, 6-MWT, readmission rate, 1-year mortality	4
				Perindopril	Perindopril, 2 mg, gradually reach target dose of 8 mg, qd		43	62 ± 11	23/20	7		
Guo	2021	China	RCT	ARNI	Sacubitril valsartan, 100 mg/tablet, 100 mg/time, bid	12	58	68.25 ± 4.30	38/20	25	NT-proBNP, LVEF, LVEDD, cardiac death, readmission rate	5
				Valsartan	Valsartan, 80 mg/tablet, 80 mg/d		58	67.70 ± 3.72	41/17	22		
Li	2021	China	RCT	ARNI	Sacubitril valsartan, the initial dose is 25 mg/d, bid, and the dose is appropriately adjusted according to the patient’s condition and tolerance, and gradually increased to 200 mg/d or even greater tolerated dose	12	103	78 (72–83)	57/46	29	Total effective rate, LVEF, FS, quality of life, readmission rate, adverse events	6
				ACEI/ARB	ACEI/ARB		100	77 (72–82)	64/36	30		
Wu	2021	China	Retrospective cohort	ARNI	Sacubitril valsartan	3	95	69.32 ± 7.72	64/31	NA	NT-proBNP, LVEF, LVEDD	5
				ACEI	ACEI		99	71.42 ± 7.69	44/44	NA		
				ARB	ARB		84	70.56 ± 6.86	54/30	NA		
Liu	2021	China	Retrospective cohort	ARNI	Sacubitril valsartan, the initial dose is 50 mg each time, bid, and it is increased by 1 time after 2–4 days until the target dose is 200 mg each time, bid, a course of 3 months, a total of 1 course of treatment	3	53	62.49 ± 6.97	31/22	NA	LVEF, LVEDD, DVST, LAD, quality of life, total effective rate, adverse cardiac events	7
				Enalapril	Enalapril, 5 mg, bid, a course of 3 months, a total of 1 course of treatment		52	62.63 ± 6.85	30/22	NA		
Zhou	2021	China	RCT	ARNI	Sacubitril valsartan, initial dose 50 mg/time, bid, dose increase every 2–4 weeks times, the maximum dose is 200 mg/time, bid	6	100	68.88 ± 10.16	64/36	39	NT-proBNP, LVEF, LVEDD, LAD, total effective rate	4
				Benazepril	Benazepril hydrochloride, starting at 2.5 mg/d and gradually increasing to 10 mg/d		100	69.81 ± 9.54	61/39	32		
Chen	2021	China	RCT	ARNI	Sacubitril valsartan, starting from 50 mg/time, bid, it is also doubled or halved every 2 weeks, and titrated to the maximum tolerated dose (target dose of 200 mg/time, bid)	12	59	60.5 ± 12.6	37/22	14	LVEF, SV, 1-year mortality, readmission rate, renal function deterioration, hyperkalemia	6
				Candesartan	Candesartan, starting from 4 mg, once a day, doubling or halving the dose every 2 weeks, and gradually titrating to the maximum tolerated dose according to the patient’s tolerance (target dose: 16 mg/time, once a day)		56	60.4 ± 12.7	38/21	19		
Ye	2022	China	Retrospective cohort	ARNI	Sacubitril valsartan, 50 mg/starting dose 25 mg, bid, gradually increasing to the target dose according to the blood pressure of the patients	12	84	62.29 ± 12.82	52/32	84	LVEF, LVEDD, readmission rate	7
				Valsartan	Valsartan, 80mg, qd		86	63.49 ± 11.61	56/30	86		

Notes: ACEI, angiotensin converting enzyme inhibitor; ARB, angiotensin receptor blockers; ARNI, angiotensin receptor-neprilysin inhibitor; HF, heart failure; HFmEF, heart failure with mid-range ejection fraction; NA, not available; RCT, randomized controlled trial; SD, standard deviation; bid, twice a day; qd, every day; CAD, coronary atherosclerotic heart disease; NT-proBNP, N-terminal-pro brainnatriuretic peptide; LVEDD, left ventricular end diastolic dimension; LVESD, left ventricular end-systolic diameter; LVEF, left ventricular ejection fraction; DVST, diastolic ventricular septal thickness; LAD, left atrial diameter; CRP, C-reactive protein; Scr, serum creatinine; SV, stroke volume; FS, left ventricular short axis shortening rate; GDF-15, growth differentiation factor-15; sST2, soluble suppression of tumorigenesis-2; 6-MWT, 6-min walking distance trial.

**TABLE 2:** Left ventricular function outcomes before and after treatment between sacubitril-valsartan and ACEI/ARB group.

Outcomes	Author	Year	Controls	Observation group			Control group		
				Sample size [n (%)]	Before treatment (mean ± SD)	After treatment (mean ± SD)	Sample size [n (%)]	Before treatment (mean ± SD)	After treatment (mean ± SD)
Left ventricular function									
LVEF (%)	Fanhao Ye	2022	Valsartan	84	44.69 ± 4.6	54.76 ± 4.24	86	44.64 ± 4.42	49.28 ± 3.47
	Yi Wu_a	2021	ACEI	95	43.23 ± 2.9	44.88 ± 7.07	99	44.05 ± 2.82	46.7 ± 5.06
	Yi Wu_b	2021	ARB	95	43.23 ± 2.9	44.88 ± 7.07	84	44.09 ± 2.67	47.09 ± 5.39
	Yi Wu_c	2021	ACEI	95	43.23 ± 2.9	45.08 ± 7.77	99	44.05 ± 2.82	49.28 ± 5.38
	Yi Wu_d	2021	ARB	95	43.23 ± 2.9	45.08 ± 7.77	84	44.09 ± 2.67	50.77 ± 6.02
	Xiangjie Liu	2021	Enalapril	53	44.23 ± 1.08	55.37 ± 4.06	52	44.29 ± 1.05	51.05 ± 3.94
	Yongyue Zhou_a	2021	Benazepril	93	44.28 ± 2.49	45.36 ± 2.67	92	44.47 ± 2.54	45.16 ± 2.85
	Yongyue Zhou_b	2021	Benazepril	93	44.28 ± 2.49	47.52 ± 2.72	92	44.47 ± 2.54	46.09 ± 2.84
	Yongyue Zhou_c	2021	Benazepril	93	44.28 ± 2.49	50.34 ± 2.71	92	44.47 ± 2.54	47.95 ± 2.63
	Guokun Wang	2019	ACEI/ARB	48	42.6 ± 2.5	52.3 ± 3.5	48	41.8 ± 3.1	46.5 ± 2.4
	Meixian Chen	2021	Candesartan	57	43.5 ± 2.1	49 ± 6	56	43.8 ± 2.3	43.8 ± 7.4
	Rong Liu	2020	Benazepril	20	44.1 ± 2.73	48.75 ± 6.77	20	44.8 ± 2.84	50.95 ± 7.42
	Xiang Li_a	2021	ACEI/ARB	103	43.82 ± 2.81	45.09 ± 2.99	100	44.03 ± 2.83	44.48 ± 3.89
	Xiang Li_b	2021	ACEI/ARB	103	43.82 ± 2.81	51.27 ± 5.18	100	44.03 ± 2.83	46.49 ± 4.2
	Xiang Li_c	2021	ACEI/ARB	103	43.82 ± 2.81	57.15 ± 4.07	100	44.03 ± 2.83	48.74 ± 3.93
	He Wen	2020	Valsartan	41	44.6 ± 3.4	51.2 ± 3.8	41	43.9 ± 1.9	49.5 ± 2.8
	Pengfei Ma	2020	Analapril	50	44.3 ± 1.2	55.41 ± 4.13	50	44.12 ± 1.17	51.19 ± 4.02
	Cunfang Chen_a	2020	ACEI/ARB	45	42.8 ± 2.3	44.7 ± 3.1	45	43.1 ± 2.2	44.2 ± 3
	Cunfang Chen_b	2020	ACEI/ARB	45	42.8 ± 2.3	47.3 ± 3.1	45	43.1 ± 2.2	45.6 ± 2.5
	Cunfang Chen_c	2020	ACEI/ARB	45	42.8 ± 2.3	52.8 ± 3.5	45	43.1 ± 2.2	46.5 ± 3.1
	Hong Mi	2021	Benazepril	30	NA ± NA	64.58 ± 8.14	30	NA ± NA	58.13 ± 7.32
	Dongrui Xu	2021	Eenalapril	49	43.9 ± 3.2	53.2 ± 4.1	49	43 ± 2.8	45.6 ± 3.4
	Xinxin Guo	2021	Valsartan	58	45.15 ± 3.9	51.2 ± 2.05	58	46.1 ± 3.35	49.05 ± 2.92
LVEDD (mm)	Fanhao Ye	2022	Valsartan	84	51.52 ± 6.2	47.26 ± 4.71	86	50.05 ± 5.62	50.12 ± 5.62
	Yi Wu_a	2021	ACEI	95	56.86 ± 7.2	55.5 ± 8.21	99	55.43 ± 6.21	52.49 ± 7.04
	Yi Wu_b	2021	ARB	95	56.86 ± 7.2	55.5 ± 8.21	84	55.46 ± 7.66	53.72 ± 7.69
	Yi Wu_c	2021	ACEI	95	56.86 ± 7.2	50.34 ± 7.34	99	55.43 ± 6.21	50.93 ± 6.65
	Yi Wu_d	2021	ARB	95	56.86 ± 7.2	50.34 ± 7.34	84	55.46 ± 7.66	50.81 ± 7.55
	Xiangjie Liu	2021	Enalapril	53	60.25 ± 5.97	47.53 ± 4.31	52	60.31 ± 5.92	52.27 ± 5.13
	Yongyue Zhou_a	2021	Benazepril	93	55.28 ± 7.14	53.89 ± 5.31	92	56.16 ± 6.98	55.19 ± 6.48
	Yongyue Zhou_b	2021	Benazepril	93	55.28 ± 7.14	51.98 ± 6.31	92	56.16 ± 6.98	54.17 ± 5.87
	Yongyue Zhou_c	2021	Benazepril	93	55.28 ± 7.14	47.84 ± 7.31	92	56.16 ± 6.98	52.14 ± 5.26
	Pengfei Ma	2020	Analapril	50	60.03 ± 6.02	47.62 ± 8.31	50	60.19 ± 6.05	52.32 ± 5.2
	Cunfang Chen_a	2020	ACEI/ARB	45	57.4 ± 9.1	55.2 ± 9.31	45	57.9 ± 8.7	57.2 ± 7.9
	Cunfang Chen_b	2020	ACEI/ARB	45	57.4 ± 9.1	51.2 ± 10.31	45	57.9 ± 8.7	56.4 ± 8
	Cunfang Chen_c	2020	ACEI/ARB	45	57.4 ± 9.1	46.7 ± 11.31	45	57.9 ± 8.7	55.4 ± 8
	Hong Mi	2021	Benazepril	30	NA ± NA	63.01 ± 12.31	30	NA ± NA	62.33 ± 6.37
	Dongrui Xu	2021	Eenalapril	49	57.9 ± 6.5	47.6 ± 13.31	49	57.8 ± 5.7	54.5 ± 6.1
	Xinxin Guo	2021	Valsartan	58	48.58 ± 4.5	46.84 ± 14.31	58	49.2 ± 4.78	48.57 ± 5.13
LVESD (mm)	Rong Liu	2020	Benazepril	20	54.15 ± 5.54	55 ± 5.36	20	54.55 ± 7.67	55.15 ± 6.06
	Hong Mi	2021	Benazepril	30	NA ± NA	41.91 ± 4.69	30	NA ± NA	42.11 ± 4.57
DVST (mm)	Xiangjie Liu	2021	Enalapril	53	12.16 ± 1.48	8.98 ± 0.86	52	12.22 ± 1.53	10.32 ± 1.07
	Pengfei Ma	2020	Analapril	50	12.27 ± 1.6	9.04 ± 0.9	50	12.2 ± 1.53	10.29 ± 1.12
	Dongrui Xu	2021	Eenalapril	49	10.1 ± 1.5	7.9 ± 1.1	49	9.4 ± 1.5	8.4 ± 1.1
LAD (mm)	Xiangjie Liu	2021	Enalapril	53	44.56 ± 4.08	34.13 ± 3.15	52	44.36 ± 4.12	36.77 ± 3.44
	Yongyue Zhou_a	2021	Benazepril	93	43.52 ± 7.69	42.36 ± 7.33	92	44.26 ± 8.39	43.83 ± 7.97
	Yongyue Zhou_b	2021	Benazepril	93	43.52 ± 7.69	40.6 ± 6.87	92	44.26 ± 8.39	43.26 ± 7.62
	Yongyue Zhou_c	2021	Benazepril	93	43.52 ± 7.69	38.83 ± 6.17	92	44.26 ± 8.39	42.66 ± 7.07
	Rong Liu	2020	Benazepril	20	41.15 ± 4.02	39.2 ± 3.68	20	40.45 ± 5.21	39.4 ± 8.41
	Pengfei Ma	2020	Analapril	50	44.29 ± 4.09	34.2 ± 3.23	50	44.63 ± 4.12	36.82 ± 3.5
	Dongrui Xu	2021	Eenalapril	49	39.6 ± 3.5	37.1 ± 3.4	49	39.3 ± 2.9	38.4 ± 2.1
SV (ml)	Meixian Chen	2021	Candesartan	57	80 ± NA	91.9 ± 15.8	56	75.9 ± NA	75.1 ± 13.5
FS (%)	Xiang Li_a	2021	ACEI/ARB	103	22.21 ± 2.73	22.67 ± 1.69	100	22.35 ± 1.86	22.46 ± 2.28
	Xiang Li_b	2021	ACEI/ARB	103	22.21 ± 2.73	26.2 ± 3.39	100	22.35 ± 1.86	23.54 ± 2.39
	Xiang Li_c	2021	ACEI/ARB	103	22.21 ± 2.73	28.47 ± 2.97	100	22.35 ± 1.86	24.51 ± 2.13
	He Wen	2020	Valsartan	41	21.8 ± 2.3	26 ± 2.6	41	21 ± 1.8	24.6 ± 1.7
SCr (μmol/L)	Guokun Wang	2019	ACEI/ARB	48	78.8 ± 6.2	95.3 ± 5.8	48	77.9 ± 7.3	86.2 ± 5.7
	Dongrui Xu	2021	Eenalapril	49	86.9 ± 32.2	92.2 ± 27.7	49	86.9 ± 34.7	104 ± 28.2
CRP (μg/ml)	Dongrui Xu	2021	Eenalapril	49	9.6 ± 7.8	3.1 ± 2.3	49	9.6 ± 8.1	4.5 ± 3.7
Indicators related to heart failure									
NT-proBNP (ng/L)	Yi Wu_a	2021	ACEI	95	3397.03 ± 4951.87	2469.07 ± 4830.57	99	3935.4 ± 4736.22	2103.18 ± 2460.54
	Yi Wu_b	2021	ARB	95	3397.03 ± 4951.87	2469.07 ± 4830.57	84	4485.76 ± 5352.09	2491.6 ± 2924.16
	Yi Wu_c	2021	ACEI	95	3397.03 ± 4951.87	1893.28 ± 4093.11	99	3935.4 ± 4736.22	1704.1 ± 2639.72
	Yi Wu_d	2021	ARB	95	3397.03 ± 4951.87	1893.28 ± 4093.11	84	4485.76 ± 5352.09	1747.12 ± 2214.61
	Yongyue Zhou_a	2021	Benazepril	93	7598.9 ± 4423.76	4202.06 ± 2282.2	92	7335.77 ± 4334.95	5040 ± 2660.5
	Yongyue Zhou_b	2021	Benazepril	93	7598.9 ± 4423.76	2193.78 ± 1150.39	92	7335.77 ± 4334.95	3456.54 ± 1709.68
	Yongyue Zhou_c	2021	Benazepril	93	7598.9 ± 4423.76	1073.11 ± 520.17	92	7335.77 ± 4334.95	2262.91 ± 1043.98
	Guokun Wang_d	2019	ACEI/ARB	48	1844.1 ± 321.1	444.2 ± 158.2	48	1835.2 ± 342.1	756.3 ± 217.7
	Rong Liu	2020	Benazepril	20	2736 ± 3277	1024 ± 908	20	2533 ± 2452	1232 ± 1083
	He Wen	2020	Valsartan	41	4151 ± 576	3129 ± 560	41	4149 ± 285	3764 ± 165
	Pengfei Ma	2020	Analapril	50	2619.8 ± 25.02	1160.51 ± 10.32	50	2623.36 ± 25.8	1400.47 ± 12.69
	Cunfang Chen_a	2020	ACEI/ARB	45	2729.4 ± 372.1	2052.9 ± 343	45	2811.8 ± 382.2	2188.2 ± 355.1
	Cunfang Chen_b	2020	ACEI/ARB	45	2729.4 ± 372.1	1164.7 ± 276	45	2811.8 ± 382.2	1852.9 ± 381
	Cunfang Chen_c	2020	ACEI/ARB	45	2729.4 ± 372.1	782.4 ± 245.5	45	2811.8 ± 382.2	1411.8 ± 340.7
	Dongrui Xu	2021	Eenalapril	49	2762.8 ± 557.9	851.2 ± 232.1	49	2812 ± 402.8	1580.8 ± 333.9
	Man Gao	2021	Perindopril	43	2334 ± 978	1313 ± 664	43	2183 ± 938	1588 ± 892
	Xinxin Guo	2021	Valsartan	58	1835.75 ± 702.36	892.35 ± 422.64	58	1786.52 ± 673.24	1107.53 ± 450.12
sST2 (pg/ml)	He Wen	2020	Valsartan	41	70 ± 10	58 ± 10	41	80 ± 9	69 ± 8
	Dongrui Xu	2021	Eenalapril	49	75.1 ± 38.5	62.8 ± 7.1	49	73.3 ± 7	66.7 ± 10.4
GDF-15 (pg/ml)	He Wen	2020	Valsartan	41	1110 ± 270	686 ± 2749	41	1015 ± 342	752 ± 303

Notes: ACEI, angiotensin converting enzyme inhibitor; ARB, angiotensin receptor blockers; LVEF: left ventricular ejection fraction; LVEDD: left ventricular end diastolic dimension; LVESD: left ventricular end-systolic diameter; DVST: diastolic ventricular septal thickness; LAD: left atrial diameter; SV: stroke volume; FS: left ventricular short axis shortening rate; CRP: C-reactive protein: NT-proBNP: N-terminal-pro brain natriuretic peptide; GDF-15:growth differentiation factor-15; sST2: soluble suppression of tumorigenesis-2. The a, b c, and d represent different controls or different duration of intervention times.

### Left ventricular function

#### LVEF (%)

**FIGURE 2:**
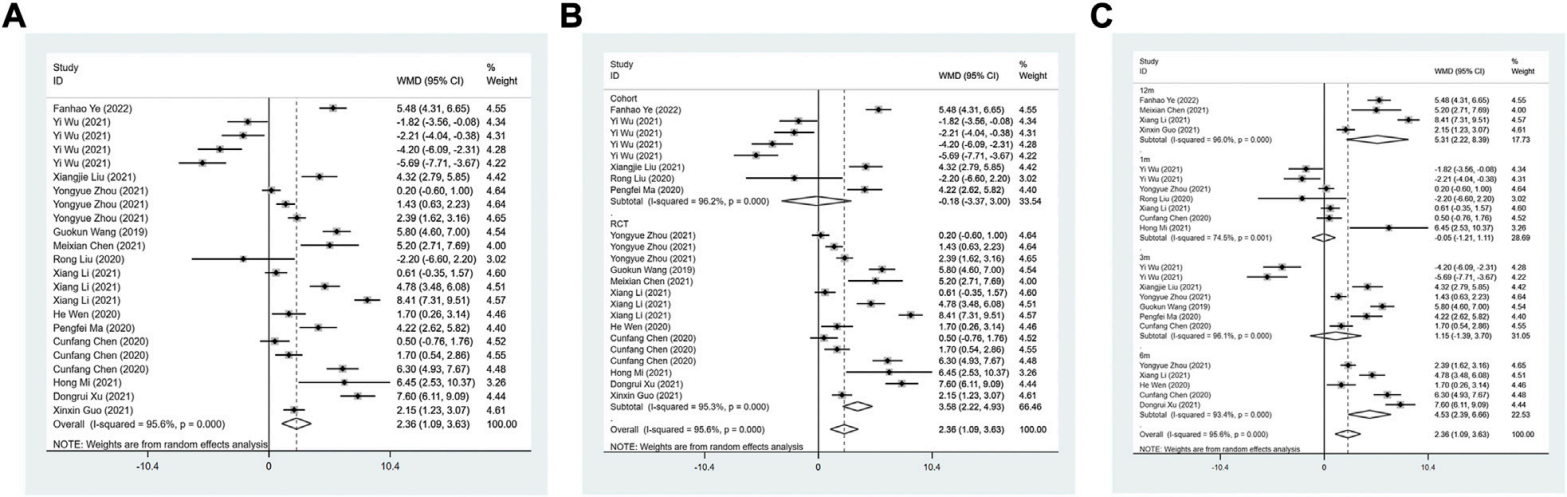
Forest plot of LVEF between sacubitril-valsartan and ACEI or ARB; **(A)** overall; **(B)** subgroup analysis of study type; **(C)** duration of treatment.

A total of 14 studies were included to evaluate the effect of ARNI on LVEF. The heterogeneity test results showed I^2^ = 95.6%, therefore, the random effect model was used. The result demonstrated that LVEF levels were significantly improved in patients with HFmrEF in the ARNI group (WMD: 2.36, 95%CI: 1.09 to 3.63, *p* < 0.001) (Table 3; Figure 2A). Subgroup analysis based on study type showed that LVEF levels were significantly higher in patients with HFmrEF in the ARNI group compared with ACEI or ARB group in the RCT study (I^2^ = 95.3%, WMD: 3.58, 95%CI: 2.22 to 4.93, *p* < 0.001) (Table 3; Figure 2B). However, in cohort studies, there was no difference in LVEF levels between the ARNI group and the ACEI or ARB group (WMD: −0.18, 95%CI: −3.37 to 3.00, *p* = 0.91). According to the duration of treatment subgroup analysis, there was no difference in LVEF between the two groups at 1 and 3 months of treatment (*p* > 0.05). At 6 and 12 months of treatment, the LVEF level of the ARNI group was higher than that of the control group, with the WMD being 4.53 (95%CI: 2.39 to 6.66, *p* < 0.001) in 6 months, and being 5.31 (95%CI: 2.22 to 8.39, *p* = 0.001) in 12 months, respectively (Table 3; Figure 2C).

**TABLE 3:** Efficacy and safety of sacubitril-valsartan treatment in HFmrEF patients.

Outcomes	WMD (95%CI)	*p*	I^2^
Left ventricular function			
LVEF (%)	2.36 (1.09, 3.63)	<0.001	95.6
Study design			
RCT	3.58 (2.22, 4.93)	<0.001	95.3
Cohort	−0.18 (−3.37, 3.00)	0.911	96.2
Duration of treatment (months)			
1	−0.05 (−1.21, 1.11)	0.927	74.5
3	1.15 (−1.39, 3.70)	0.374	96.1
6	4.53 (2.39, 6.66)	<0.001	93.4
12	5.31 (2.22, 8.3)	0.001	96
LVEDD (mm)	−2.48 (−3.83, −1.13)	<0.001	84.9
Study design			
RCT	−3.45 (−5.11, −1.80)	<0.001	79.3
Cohort	−1.29 (−3.46, 0.89)	0.247	88.5
Duration of treatment (months)			
1	0.52 (−1.41, 2.46)	0.595	66.8
3	−2.90 (−4.58, −1.22)	0.001	75.1
6	−6.34 (−8.88, −3.81)	<0.001	73.6
12	−2.37 (−3.54, −1.20)	<0.001	0
LVESD (mm)	−0.19 (−2.14, 1.77)	0.853	0
DVST (mm)	−2.03 (−4.39, 0.33)	0.091	99
Study design			
RCT	−0.50 (−0.94, −0.06)	0.024	NA
Cohort	−2.80 (−5.82, 0.23)	0.07	99.2
LAD (mm)	−2.23 (−2.83, −1.6)	<0.001	21.8
FS (%)	2.054 (0.25, 3.86)	0.025	95.9
Duration of treatment (months)			
1	0.21 (−0.34, 0.76)	0.457	NA
6	2.06(0.82, 3.29)	0.001	74.5
12	3.96 (3.25, 4.67)	<0.001	NA
Indicators related to heart failure			
NT-proBNP (ng/L)	−494.92 (−641.34, −348.50)	<0.001	93.9
Study design			
RCT	−623.33 (−812.19, −434.47)	<0.001	93.3
Cohort	−239.93 (−244.47, −235.40)	<0.001	0
Duration of treatment (months)			
1	−181.53 (−403.67, 40.62)	0.109	12
3	−467.60 (−664.02, −271.17)	<0.001	92.7
6	−774.37 (−961.72, −587.03)	<0.001	83.3
12	−226.31 (-369.67, −82.95)	0.002	0
sST2 (pg/ml)	−7.40 (−14.35, −0.44)	0.037	85.6
Quality of life score			
MHFQL	−10.13 (−20.61, 0.34)	0.058	98.4
Study design			
RCT	−0.70 (−2.30, 0.90)	0.39	NA
Cohort	−14.92 (−16.79, −13.04)	<0.001	0
KCCQ	4.13 (3.46, 4.81)	<0.001	0
6-MWT (m)	51.35 (26.99, 75.71)	<0.001	5.1
Total effective rate	1.15 (1.08, 1.21)	<0.001	0
Mortality			
1-year mortality	0.69 (0.38, 1.27)	0.23	0
Cardiac death	0.62 (0.26, 1.46)	0.272	0
Readmission rate	0.54 (0.43, 0.68)	<0.001	0
Adverse events			
Renal function deterioration	0.57 (0.26, 1.23)	0.151	0
Hypotension	0.71 (0.36, 1.39)	0.311	0
SCr (μmol/L)	−0.62 (−21.05, 19.81)	0.953	92.4
Duration of treatment (months)			
3	9.10 (6.80, 11.40)	<0.001	NA
6	−11.80 (−22.87, −0.73)	0.037	NA

Notes: HFmrEF: heart failure with mid-range ejection fractions; WMD: weighted mean difference; RR: relative risk; CI: confidence interval; LVEF: left ventricular ejection fraction; LVEDD: left ventricular end diastolic dimension; LVESD: left ventricular end-systolic diameter; DVST: diastolic ventricular septal thickness; LAD: left atrial diameter; SV: stroke volume; FS: left ventricular short axis shortening rate; CRP: C-reactive protein: NT-proBNP: N-terminal-pro brain natriuretic peptide; sST2: soluble suppression of tumorigenesis-2; MHFQL: Minnesota heart failure scale score; KCCQ: The kansas city cardiomyopathy questionnaire; 6-MWT: 6-Minute Walk Test; SCr: serum creatinine.

#### LVEDD (mm)

**FIGURE 3:**
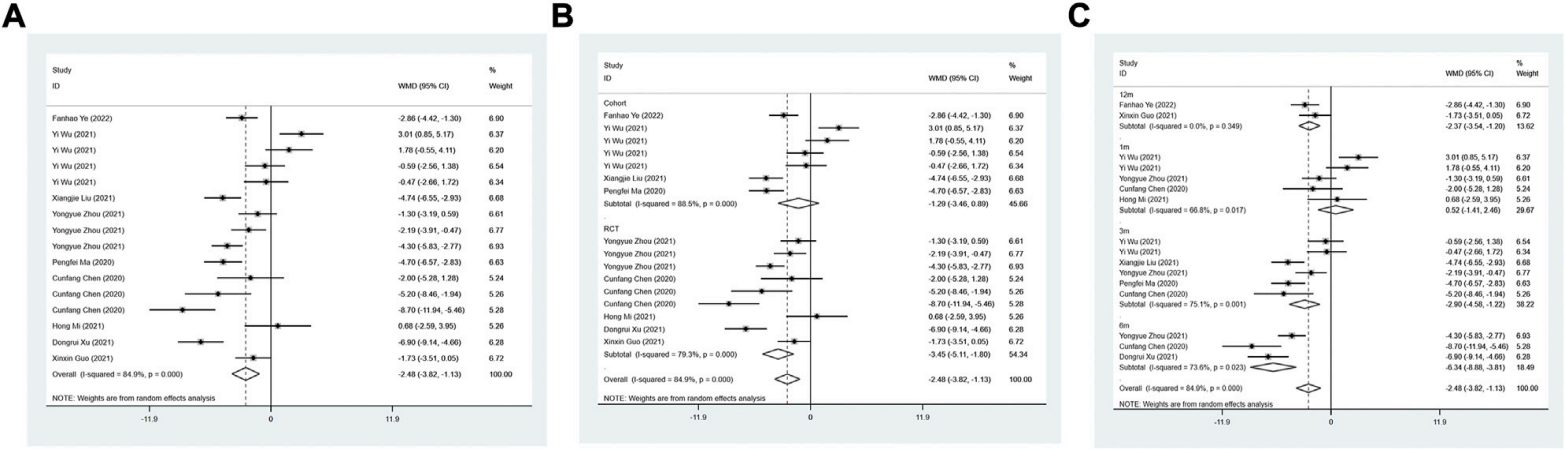
Forest plot of LVEDD between sacubitril-valsartan and ACEI or ARB; **(A)** overall; **(B)** subgroup analysis of study type; **(C)** duration of treatment.

The LVEDD between the ARNI group and ACEI or ARB group was assessed in 9 studies. The random effect model results suggested that LVEDD in ARNI group was lower than that in the control group after treatment (WMD: −2.48, 95%CI: −3.83 to −1.13, *p* < 0.001) (Table 3; Figure 3A). Subgroup results based on RCTs also showed a similar result (I^2^ = 79.3%, WMD: −3.45, 95%CI: −5.11 to −1.80, *p* < 0.001) (Table 3; Figure 3B). In term of the subgroup analysis of duration of treatment, there was no difference in LVEDD between the two groups after 1 month of treatment (*p* > 0.05). At 3 (WMD: −2.90, 95%CI: −4.58 to −1.22, *p* = 0.001), 6 (WMD: −6.34, 95%CI: −8.88 to −3.81, *p* < 0.001) and 12 months of treatment (WMD:−2.37, 95%CI: −3.54 to −1.20, *p* < 0.001), LVEDD in the ARNI group was lower than that in the control group (Table 3; Figure 3C).

#### LVESD (mm)

A total of two studies were included to evaluate the LVESD after treatment between ARNI and ACEI or ARB groups. The fixed-effect model indicated no statistically significant difference in LVESD between ARNI and ACEI or ARB treatment (WMD: −0.19, 95%CI: −2.14 to 1.77, *p* = 0.85) (Table 3).

#### DVST (mm)

A total of 3 studies assessed DVST after treatment. The random-effect model indicated ARNI was not superior to ACEI or ARB treatment in DVST (I^2^ = 99%, WMD: −2.03, 95%CI: −4.39 to 0.33, *p* = 0.091). However, according to the study type analysis, DVST was lower in the ARNI group than ACEI or ARB group. (WMD: −0.50, 95%CI: −0.94 to −0.06, *p* = 0.024) (Table 3).

#### LAD (mm)

**FIGURE 4:**
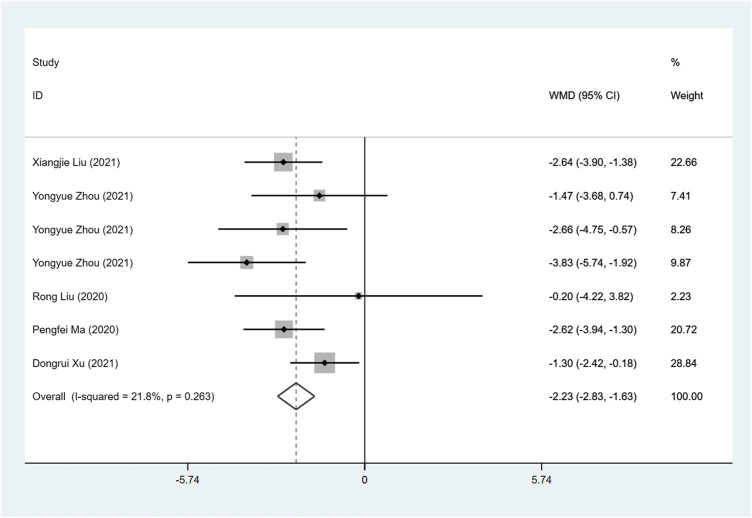
Forest plot of LAD between sacubitril-valsartan and ACEI or ARB.

Five studies investigate LAD between ARNI and ACEI or ARB treatment. ARNI showed a greater decrease in LAD (I^2^ = 21.8%, WMD: −2.23, 95%CI: −2.83 to −1.63, *p* < 0.001) (Table 3; Figure 4).

#### SV (ml)

Meixian Chen et al. (2021) studied the effect of ARNI vs. candesartan on stroke output of patients, and the results showed that SV in the experimental group was higher than that in the control group (WMD: 16.80, 95%CI: 11.39 to 22.22, *p* < 0.001).

#### FS (%)

**FIGURE 5:**
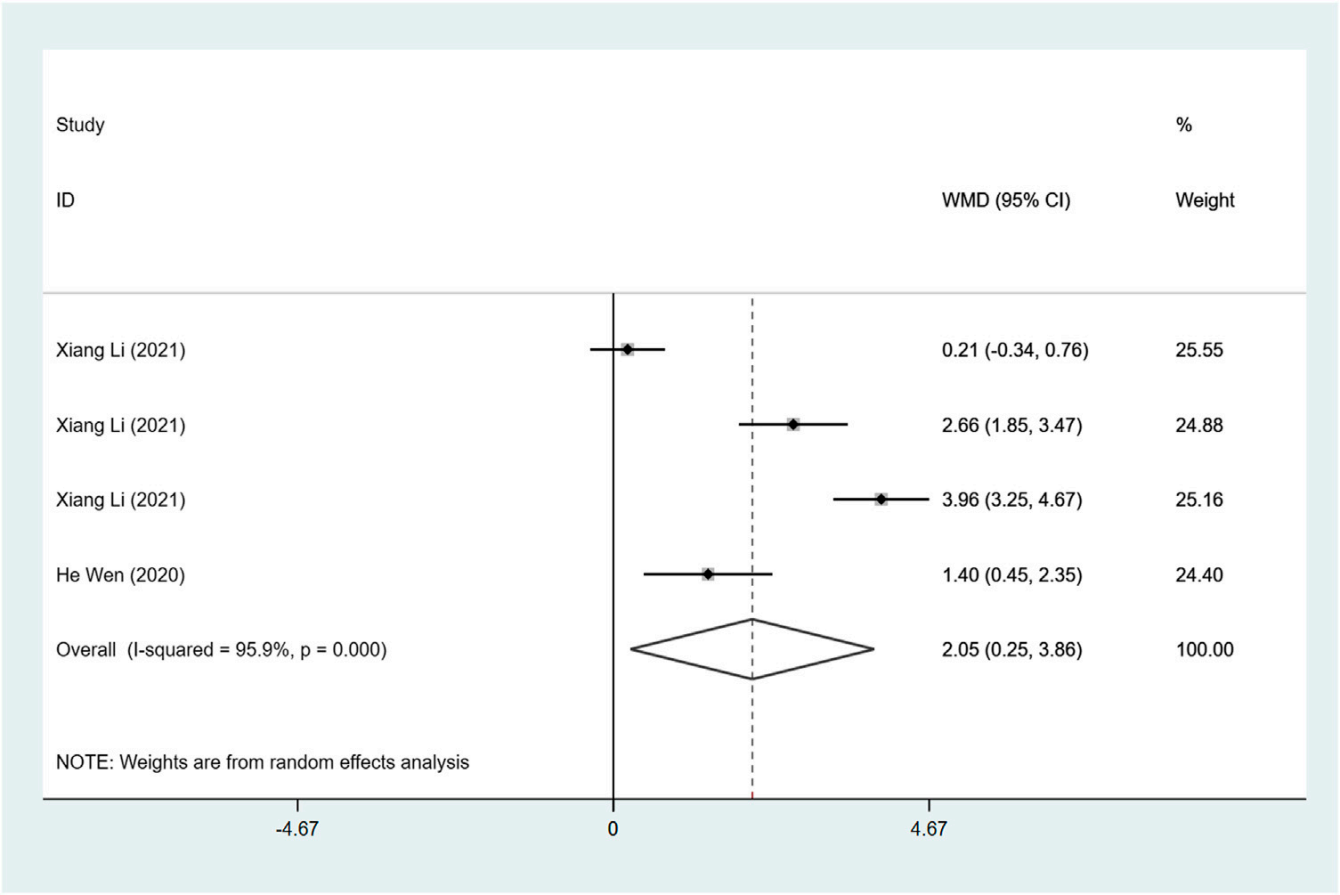
Forest plot of FS between sacubitril-valsartan and ACEI or ARB.

Two articles were included to investigate FS, and the difference in heterogeneity test results showed I^2^ = 95.9%, so the random effect model was used for analysis. The result demonstrated that FS after ARNI treatment was higher than ACEI or ARB treatment (WMD: 2.05, 95%CI: 0.25 to 3.86, *p* = 0.025) (Table 3; Figure 5).

#### CRP (μg/ml)

A study Dongruil Xu and Liu (2021) by Xu et al. investigated the effect of ARNI VS enalapril on CRP in HFmrEF patients. The results indicated that CRP in the ARNI group was lower than that in the control group (WMD: −1.40, 95%CI: −2.62 to −0.18, *p* = 0.024).

### Indicators related to HF

#### NT-proBNP (ng/L)

**FIGURE 6:**
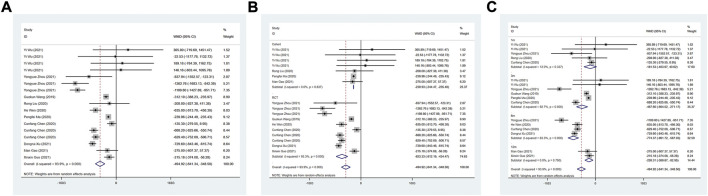
Forest plot of NT-proBNP between sacubitril-valsartan and ACEI or ARB; **(A)** overall; **(B)** subgroup analysis of study type; **(C)** duration of treatment.

Ten studies were used to investigate the NT-proBNP level after treatment. A random effect model was used for analysis. ARNI could significantly reduce patients’ NT-proBNP (WMD: −494.92, 95%CI: −641.34 to −348.50, *p* < 0.001) (Table 3 Figure 6A). Based on the subgroup analysis of study type, whether in RCT study (I^2^ = 93.3%, WMD: −623.33, 95%CI: −812.19 to −434.47, *p* < 0.001) or cohort study (I^2^ = 0.0%, WMD: −239.93, 95%CI: −244.47, −235.40, *p* < 0.001), patients treated by ARNI had lower NT-proBNP (Table 3; Figure 6B). According to the subgroup analysis of the duration of treatment, there was no statistical difference in NT-proBNP between the two groups (*p* > 0.05) after 1 month of treatment. At 3 (WMD: −467.60, 95%CI: −664.02 to −271.17, *p* < 0.001), 6 (WMD: −774.37, 95%CI: −961.72 to −587.03, *p* < 0.001) and 12 months (WMD: −226.31, 95%CI: −369.67 to −82.95, *p* = 0.002) of treatment, nT-proBNP in the ARNI group was lower than that in the control group (Table 3; Figure 6C).

#### sST2 (pg/ml)

**FIGURE 7:**
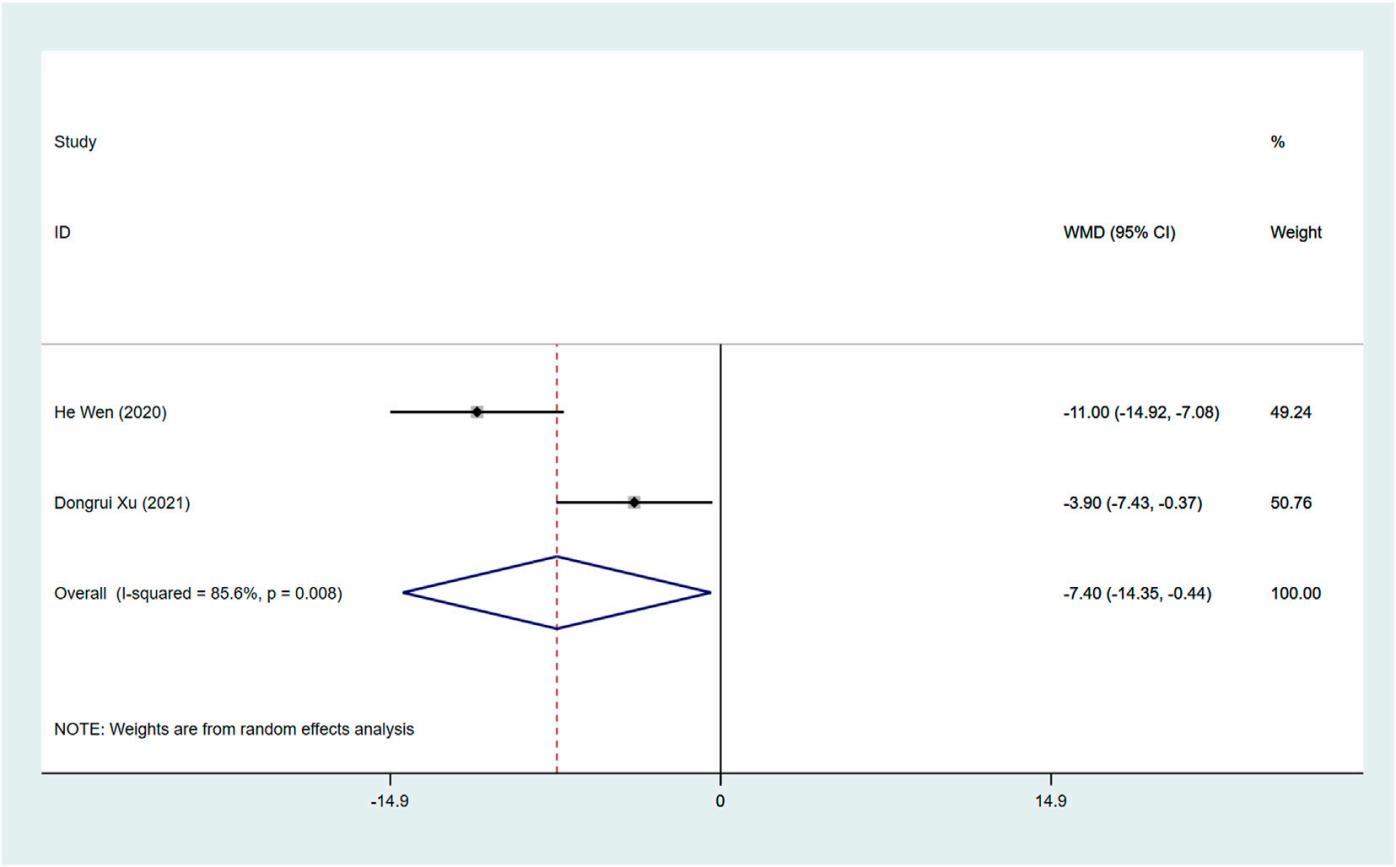
Forest plot of sST2 between sacubitril-valsartan and ACEI or ARB.

Two studies assessed the sST2 after treatment between ARNI and ACEI or ARB groups. The random effect model analysis showed that sST2 in the ARNI group was lower than that in ACEI or ARB group (WMD: −7.40, 95%CI: −14.35 to −0.44, *p* = 0.037) (Table 3; Figure 7).

#### GDF-15 (pg/ml)

He Wen et al. (2020) studied the effect of ARNI vs valsartan on the growth and transformation factor of GDF-15 in HFmrEF patients. The results showed no statistical difference between the two groups of GDF-15 (WMD: −66.00, 95%CI: −912.55 to 780.55, *p* = 0.879).

### Quality of life score

#### MHFQL

Three studies used MHFQL to evaluate the effects of ARNI and ACEI or ARB on quality of life. Random effects model analysis showed no difference in MHFQL between the two groups (WMD: −10.13, 95%CI: −20.61 to 0.34, *p* = 0.058) (Table 3).

#### KCCQ

A total of 1 study containing 3 groups of data were included to assess quality of life through KCCQ. A higher quality of life was found in HFmrEF patients using ARNI (WMD: 4.13, 95%CI: 3.46 to 4.81, *p* < 0.001) (Table 3).

#### 6-MWT (m)

Three articles were included to assess 6-MWT. The fixed-effect model results showed that the treatment of ARNI significantly increased 6-MWT in HF patients with HFmrEF (I^2^ = 5.1%, WMD: 51.35, 95%CI: 26.99 to 75.71, *p* < 0.001) (Table 3).

### Total effective rate

Total effective rate was evaluated in 6 studies, and the heterogeneity test results indicated I^2^ = 0.0%, so fixed-effect model was used. The total effective rate of ARNI was higher than that of ACEI or ARB (RR: 1.15, 95%CI: 1.08 to 1.21, *p* < 0.001) (Table 3).

### Mortality

#### 1-year mortality

The 1-year mortality was assessed in 3 studies. We use the fixed-effect model to combine analysis, and the results showed that there was no difference in 1-year mortality between the ARNI and ACEI or ARB treatments groups (RR: 0.69, 95%CI: 0.38 to 1.27, *p* = 0.230), with the 1-year mortality rate being 0.09 in the ARNI group and 0.18 in the ACEI or ARB group (Tables 2, 3).

#### Cardiac death

Four studies evaluated cardiac death after the ARNI and ACEI or ARB treatments. The fixed-effect model results showed the ARNI was no better than ACEI or ARB in decreasing cardiac death (RR: 0.62, 95%CI: 0.26 to 1.46, *p* = 0.272). The incidence of cardiac death was 0.04 after the ARNI treatment and 0.06 in the ACEI or ARB treatment (Table 3).

### Readmission rate

**FIGURE 8:**
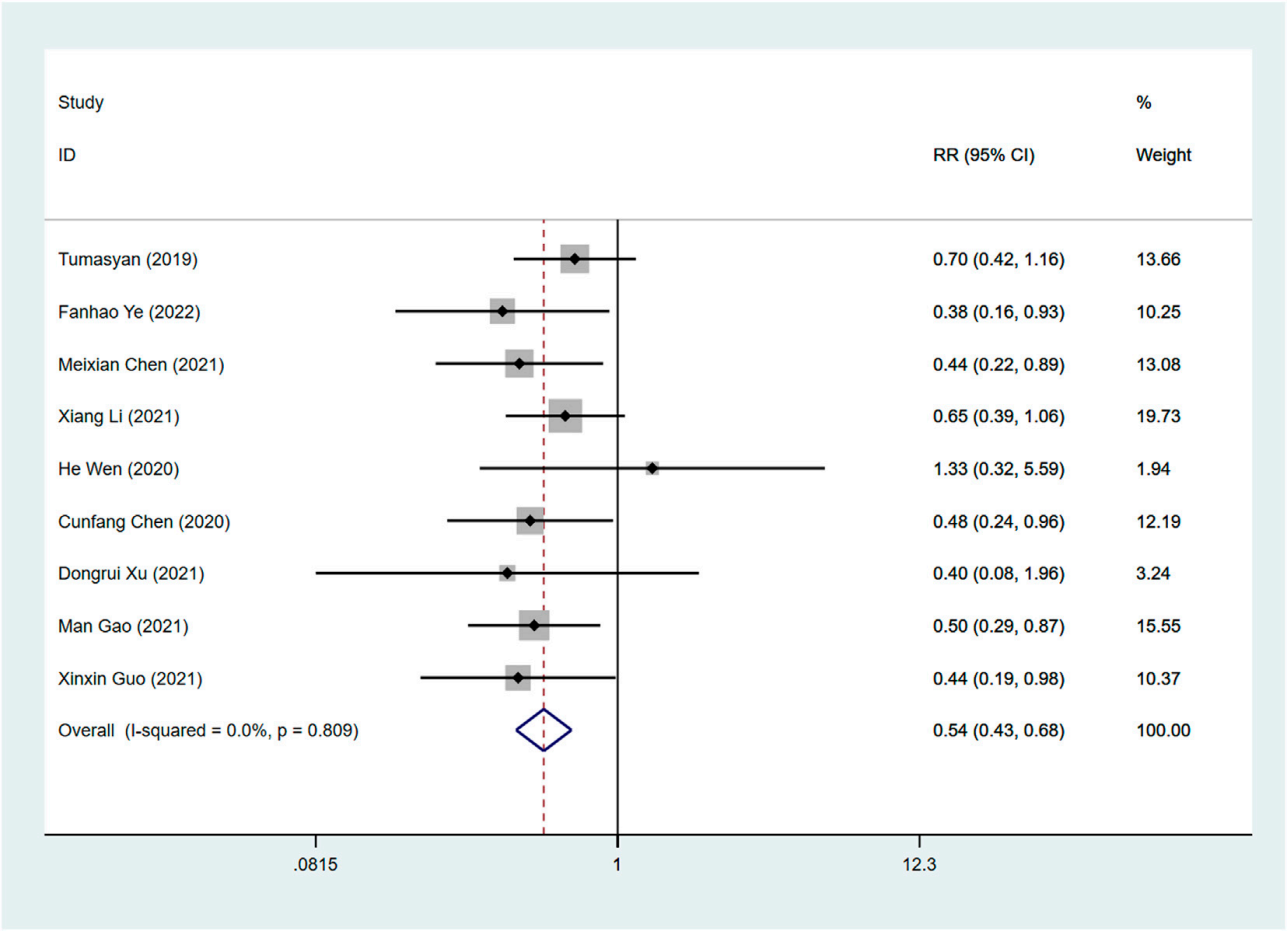
Forest plot of readmission rate between sacubitril-valsartan and ACEI or ARB.

A total of 9 articles examined the effects of ARNI and ACEI or ARB on readmission rates. Our combined results indicated that the use of ARNI was associated with a greater reduction in readmission rates than the ACEI or ARB (RR: 0.54, 95%CI: 0.43 to 0.68, *p* < 0.001) (Table 3; Figure 8).

### Adverse events

#### Worsening HF and malignant arrhythmia

Xiangjie Liu (2021) evaluated the effects of ARNI on cardiac function and short-term prognosis in HFmrEF patients and found that the incidence of worsening HF and malignant arrhythmia in ARNI was not statistically significant compared with enalapril maleate tablets in the control group.

#### Hyperkalemia

A study by Meixian Chen et al. (2021) found that the incidence of hyperkalemia in ARNI and the control group was not statistically significant.

#### Angioedema

A clinical study (Xiang Li, 2021) evaluating the efficacy and safety of ARNI in the treatment of patients with HFmrEF has reported that the incidence of angioedema in ARNI was not statistically significant compared with the control group.

#### Renal function deterioration

Four studies evaluated renal function deterioration. The result demonstrated that the incidence of renal function deterioration between ARNI treatment and ACEI or ARB treatment was not significantly different (RR: 0.57, 95%CI: 0.26 to 1.23, *p* = 0.151) (Table 3).

#### Hypotension

The hypotension was assessed in 2 articles. Our result indicated no difference between ARNI treatment and ACEI or ARB treatment in hypotension (RR: 0.71, 95%CI: 0.36 to 1.39, *p* = 0.31) (Table 3).

#### SCr (μmol/L)

SCr was assessed before and after treatment in two studies, and the results of the random-effect model showed no difference in SCr between the ARNI and ACEI or ARB groups after treatment (I^2^ = 92.4%, WMD: −0.62, 95%CI: −21.05 to 19.81, *p* = 0.953) (Table 3).

### Sensitivity analysis and publication bias

Sensitivity analysis was performed through sequentially excluded individual studies to assess the stability of the results. The sensitivity analysis result demonstrated that our findings are trustworthy. Begg’s test was used to evaluate the publication bias for outcomes with ≥9 articles. According to the results, LVEF (Z = 0.74, *p* = 0.460), LVEDD (Z = 0.86, *p* = 0.392), NT-proBNP (Z = 1.44, *p* = 0.149), readmission rate (Z = 0.52, *p* = 0.602) did not have publication bias.

## Discussion

Unlike HFrEF patients, the treatment for HFmrEF patients was still symptom-based and empiric, without definitive strategies for this entity (Nie et al., 2021). Despite previous studies finding that ACEI and ARB might improve the symptoms and functional capacity of the non-HFrEF patient, they did not reduce morbidity and mortality (Solomon et al., 2012). This meta-analysis evaluating the effects of ARNI on HFmrEF patients compared with ACEI/ARB drugs demonstrated that compared with ACEI or ARB, ARNI was likely to improve left ventricular function by increasing the LVEF, SV, and FS, decreasing LVEDD, LAD, CRP, AND NT-proBNP. The ARNI had a higher total effective rate and KCCQ and 6-MWT. In addition, ARNI decreased the readmission rate.

ARNI, which consists of the neprilysin inhibitor sacubitril (AHU377) and the ARB valsartan (Tillman et al., 2019), is the first drug indicated to be superior to enalapril in reducing mortality for patients with HF and shows the potential to improve the left ventricular function of patients with HF (Solomon et al., 2016). In this study, compared with ACEI or ARB, ARNI could significantly increase the LVEF and FS, with a decreasing NT-proBNP. LVEF and FS reflect left ventricular systolic function, while NT-proBNP can reflect the ventricular volume and ventricular wall tension, both of which can evaluate the severity of HF (Newton et al., 2009). The increase of NT-proBNP and the decrease of LVEF and FS could reflect the increase in adverse events among patients with HF (Vaskova et al., 2020). A study (Xiang Li, 2021) by Li et al. showed that with the prolongation of treatment time, the NT-proBNP of the two groups showed a gradual downward trend, while the LVEF and FS showed an upward trend, however, the effect of the ARNI group was more obvious. The reduction in NT-ProBNP may indirectly reflect that ARNI could rapidly decrease the left ventricular pressure and volume overloads and improve the left ventricular function in HFmrEF patients compared with ACEI or ARB (Nie et al., 2021). sST-2 is produced by cardiomyocytes and fibroblasts when they are in a state of stress or injury and can be derived from large blood vessels and myocardial microvascular endothelial cells (Pascual-Figal and Januzzi, 2015). sST-2 can early predict myocardial fibrosis and ventricular remodeling and is an independent predictor of the prognosis of HF (Gaggin et al., 2014). The decreased degree of sST-2 in the ARNI group was more obvious than that in the control group after treatment, indicating that ARNI had obvious advantages in reducing the severity of HFmrEF (Dongruil Xu and Liu, 2021). ARNI has the dual effect of inhibiting enkephalinase and ARB (Wachter et al., 2020). On the one hand, ARNI can inhibit enkephalinase and increase peptides (such as natriuretic peptides) degraded by enkephalinase level, play the role of vasodilator, diuresis, and natriuresis, increase the cardiovascular protective effect of natriuretic peptide, inhibit myocardial hypertrophy and fibrosis, reduce cardiac load, and finally improve cardiac function (Gu et al., 2010); on the other hand, ARNI can improve hemodynamics, reduce aldosterone levels, and inhibit ventricular remodeling by inhibiting the renin-angiotensin-aldosterone system (RAAS) (Burke et al., 2019). The two mechanisms of action are complementary and overlapping and play the role of improving the left ventricular function (Albert et al., 2019).

From our study, there was no statistical difference in the incidence of adverse outcomes between the ARNI group and the ACEI/ARB group. Fröb et al. focused on the efficacy and safety of ARNI in an outpatient setting and found that ARNI improved LVEF, NT-probNP levels, and hospitalization rates, mostly without associated side effects (Fröb et al., 2022). Although ARNI shows a potential benefit to improve the left ventricular function of patients with HF, treatment with ARNI has also been reported to be associated with a higher rate of symptomatic hypotension (Zhang et al., 2020). Even though we did not find an increase in hypotension with ARNI, the safety of ARNI still needs more RCTs to be evaluated. The mortality of HFmrEF patients is another matter of concern. Hobbs et al. (2007) showed that the 5-year mortality rate of HFmrEF patients was as high as 26%. Although our findings showed that there was no difference in reducing mortality between ARNI and traditional anti-HF drugs, the 1-year mortality rate was significantly lower with ARNI than with ACEI/ARB. In a double-blind trial, ARNI was effective in reducing cardiovascular mortality in patients with HFrEF (McMurray et al., 2014). In conclusion, compared with ACEI/ARB drugs, ARNI is not worse in terms of safety in HFmrEF patients.

Quality of life is another key criterion to assess the treatment effect on HF patients. A previous study suggested that the proportion of patients with an improvement of 5 points in the KCCQ score was higher in the ARNI group (Solomon et al., 2019), which is consistent with our findings. The 6 MWT, a method to detect functional compensatory ability, is widely used in the clinical evaluation of cardiopulmonary diseases before and after therapeutic intervention (Lin et al., 2021). Our study demonstrated that the ARNI had a higher 6-MWT compared with ACEI/ARB. A study assessing the early effects of ARNI on exercise tolerance in patients with HFmHF found that ARNI improved exercise tolerance, peak VO₂, and ventilatory efficiency at 6.2 months of follow-up (Vitale et al., 2019). Giallauria et al. found that ARNI therapy improves autonomic function, functional capacity, and ventilation (Giallauria et al., 2020). In clinical practice, ARNI can be considered a drug for HFmrEF patients, however, further studies are needed to better elucidate the underlying mechanisms of this functional improvement. Despite the efficacy and safety of ARNI in the treatment of patients with HF, a study (Oh et al., 2022) by Oh et al. dementated that earlier use of ARNI was related to better clinical outcomes and earlier left ventricular reverse remodeling; remodeling of left atrial was less prominent in the later use group implying delayed response in diastolic function. The timing of initiation of ARNI therapy in patients with HF needs further investigation.

The use of ARNI in cardiac devices treated patients has also been investigated. The study by Sardu et al. (2022) evaluating the effects of ARNI in cardiac resynchronization therapy with defibrillator (CRTd) non-responders found that at 1 year of follow-up, ARNI-users had a higher increase of LVE Fand 6 MWT along with a more significant reduction of left ventricular end-systolic volume (LVESv) compared to non-ARNI users. This evidence implied that ARNI-based therapies increase the probability of anti-remodeling effects of CRTd. In addition, the study by Sardu et al. (2018) found that ST2 protein may be used as valid monitoring biomarker, and as a predictive biomarker in failing heart internal cardioverter defibrillator (ICD) patients affected by metabolic syndrome. More studies are needed to explore the effect of ARNI on the use of cardiac devices in patients with HFmHF.

Financial status may be particularly important given the high cost of the newer therapy ARNI approach; the high prevalence of geriatric diseases in elderly patients with HF, caregiver support may be particularly important in an era of the increasing complexity of pharmacologic regimens (Sumarsono et al., 2020). In addition, the elderly HF population is highly heterogeneous, with different pathophysiological mechanisms, the frequent presence of other chronic diseases, and functional and cognitive impairments that can significantly affect the utility and value of diagnostic research and therapeutic interventions (Francis et al., 2014; Gorodeski et al., 2018). However, most of the included studies were lacking in information on patients’ backgrounds, future studies still need to examine the use of ARNI in old patients with HFmrEF.

There are several limitations to this meta-analysis. First, the results should be interpreted with caution given the limited number of included RCTs, and the small sample size. Second, due to the limitation of the included literature, we could not analyze the patients with a history of chronic diseases and drug history, which might have produced confounding bias for the evaluation. Third, in order to have a better understanding of the long-term benefit and potential side effects of ARNI on HFmrEF patients, longer duration studies are needed. Future RCTs with larger sample sizes and longer-duration are needed to confirm our findings.

## Conclusion

This study evaluated the effects of ARNI on HFmrEF patients compared with ACEI/ARB drugs, and found that ARNI may be an effective and safe strategy with which to improve the left ventricular function and quality of life, reduce readmission rate in HFmEF. In the future, more well-designed trials are needed to confirm these findings and investigate whether ARNI has a clear benefit in patients with HFmrEF.
